# Hernia U: challenges and opportunities of an online platform for surgical education

**DOI:** 10.1590/0100-6991e-20202873

**Published:** 2021-03-23

**Authors:** DIEGO LAURENTINO LIMA, RAQUEL NOGUEIRA C LAURENTINO LIMA, Eduardo Parra-Davila, Salvador Morales-Conde, Flavio Malcher

**Affiliations:** 1 - Montefiore Medical Center, Department of Surgery - The Bronx - NY - Estados Unidos; 2 - Faculdade Pernambucana de Saúde, Curso de Medicina - Recife - PE - Brasil; 3 - Good Samaritan Medical Center-TENET Health, Hernia and Abdominal Wall Reconstruction - West Palm Beach - FL - Estados Unidos; 4 - Hospital Quironsalud Sagrado Corazon, General and Digestive Surgery Unit - Sevilla - Sevilla - Espanha; 5 - Montefiore Medical Center, Director Abdominal Wall Program, Department of Surgery - The Bronx - NY - Estados Unidos

The internet has become an essential tool for education^1^. Nowadays, it is widely used by physicians for obtaining medical information. There was no website for surgical education before the year 2000. The pioneer website was WebSurg, from IRCAD, France^2^. There are different types of distant education: telesurgery (live or edited), live lectures, case discussions and so on^2^ ^,^ ^3^. These new learning methods are considered as distant education and can be integrated in the surgical curriculum^2^. There are many online tools used to share knowledge: websites, mobile programs for cell phones or even social media^4^ ^,^ ^5^. The aim of this study is to describe an online and free surgical education tool for students, residents and surgeons who want to update their knowledge in abdominal wall surgery.

## Hernia U structure

Hernia U (www.herniau.com) was created with the objective to expand the abdominal wall surgery (AWS) educational landscape and make it available for surgeons all around the globe. It is an online platform where surgeons can register with no cost and subscribe for different activities: basic and deeper discussions regarding complex situations, live case reviews, a video library and podcast series.

In 2016, it was understood that it was time to expand the hernia educational landscape. Hernia specialists created the Hernia U platform to offer content to everyone who wanted to revisit their hernia education. 

## Hernia U offers the following activities:

Hernia A to Z Fundamentals: This course covers the basics of hernia repair: applied abdominal wall anatomy, techniques in inguinal hernia repair and avoiding intraoperative and postoperative complications. After completing the lectures, there is a quiz and a live question and answer chat session with all the faculty.. The course is available in English and Spanish, with Chinese, French and Arabic subtitles. 

 Hernia A to Z Advanced: A more detailed course for more experienced surgeons. It has 5 sessions, available in English or with Spanish subtitles. Each session has a forum where surgeons can ask questions and interact with the faculty. This course also offers 2 live surgery broadcasts. 

Live Surgery Webcasts: This section is composed of live surgery broadcasting, lectures, meetings and case discussions. Two surgical cases from two global leaders at two different locations around the world are broadcasted on a monthly basis. 

Library: The Hernia U library is a depository for high quality lectures, cases and the Hernia U podcasts where the globally renowned experts on the subject discuss why they do what they do. 

It is important to mention that all these tools and courses are free of charge. The participant creates a free account on the Hernia U website (herniau.com), and can join any course, program or tool at his/her convenience. Currently, more than 15,000 professionals from 157 countries have already participated in one of the available courses in the platform. Eight-hundred and fifty-nine surgeons participated in Hernia A to Z Advanced course in 2019 and 1,191 in the Fundamentals course, in 2020. Six-hundred and seventy-seven took the course in one of the other offered language: Chinese, Spanish, French or Arabic. Over its history, 7,234 surgeons have watched a Live surgery broadcast and there have been 3,465 downloads of the 8 podcasts. Lastly, 12,011 viewers have participated in the video library. 

## Social media and Surgical Education

Physicians and students are using social media and other online platforms similar to Hernia U to disseminate and to acquire knowledge. Closed groups on Facebook such as: Robotic Surgery Collaboration, International Hernia Collaboration, Mini Friends, and eight SAGES groups stimulate the discussion of patient management, surgical technique, and current medical literature regarding surgical themes^6^ ^-^ ^8^. Hernia U partners with Facebook groups such as International Hernia Collaboration (IHC) and Robotic Surgery Collaboration (RSC) for key events as Q&A sessions and broadcast. Many studies address the importance of how to evaluate the wide variety of videos and teaching material available online^9^ ^-^ ^11^. The real time discussions, the possibility of instant feedback, the audiovisual tools as videos with narration and pictures make these groups popular among surgeons, residents and medical students searching for help. The lack of peer-review processes is a potential disadvantage, and the member should carefully evaluate who is providing the information. Obviously, when using a website platform with well-known national and international surgeons supported by societies, this is not an issue. Even in platforms such as WebSurg, there is a concern with the quality of the published material, as demonstrated by Kartal et al. (2019)^11^. 

## Current challenges of Surgical Education online

It is important to highlight that only medical professionals can access the Hernia U platform. There is a broad debate regarding ethic issues regarding the dissemination of surgical cases online or live broadcast operations. The identification of the patient should always be preserved, and images or videos should not show any kind of identification that could possibly compromise patients, following HIPPA protocols^12^. 

Thinking about this issue, SAGES published a statement for clinical education and consultation when related to social media^13^. The society endorses the professional use of these social media groups for medical and surgical education and quality improvement. Furthermore, it recognizes that the participants can acquire a superior education with access to international experts. This is the basis of Hernia U: access to hernia specialists to improve how you treat your patients with simple or complex abdominal wall defects. SAGES also endorses that the surgeon should determine the need to seek patient permission when posting clinical cases, and an optional consent template was proposed^13^.

## Surgical education going live

Another popular tool to disseminate knowledge and rich discussions is the live surgery broadcast. This was possible with the development of minimally invasive surgery with high quality image and also faster internet connection around the globe^3^. It is well known that the learning curve of MIS and robotic surgery is longer than with other procedures^14^ ^,^ ^15^. Furthermore, this learning curve influences clinical and financial outcomes^16^ ^,^ ^17^. That is the reason why a live broadcast is so important: to optimize outcomes during the learning curves for the ones who are not experienced and don´t have an available high level training opportunity. However, a broad adoption of telementoring and telesurgery is limited by legal and ethics concerns. In a live broadcast procedure, the patient is exposed to previously unseen risks such as technical failure of the robot or network and hacking^11^. Therefore, it is extremely important to have a specific informed consent aligned with the Institutional Review Board before performing this modality. A key point is surgeon´s selection. Experts used to perform this modality should carefully be selected and paired with an experienced moderator who plays a crucial role, filtering the chat and flow of questions, allowing the surgeon to safely navigate through the procedure. Furthermore, patients’ safety must always come first, and Hernia U only invites surgeons who feel comfortable with this activity and follow the Ethics Committee guidelines in every country where the procedures are performed. 

In times when students and physicians look for online material to improve their knowledge regarding different types of procedures, a free platform with known and certified hernia specialists brings a solid contribution to spread a valid knowledge to improve management of patients, and an updated surgical technique based on scientific evidence.

**Figure 1:**
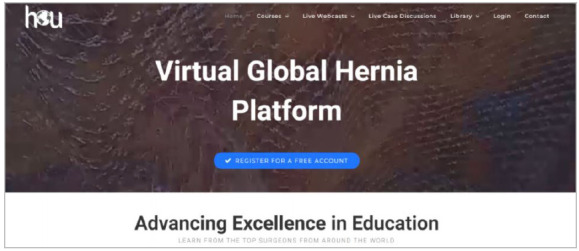
Hernia U website interface.

## References

[1] Demartines N, Mutter D, Vix M, Leroy J, Glatz D, Rösel F (2000). Assessment of Telemedicine in Surgical Education and Patient Care. Ann Surg.

[2] Mutter D, Vix M, Dallemagne B, Perretta S, Leroy J, Marescaux J (2011). WeBSurg An innovative educational Web site in minimally invasive surgery--principles and results. Surg Innov.

[3] Demartines N, Mutter D, Vix M, Leroy J, Glatz D, Rösel F (2000). Assessment of Telemedicine in Surgical Education and Patient Care. Ann Surg.

[4] Ferhatoglu MF, Kartal A, Filiz AI, Kebudi A (2019). Comparison of New Era's Education Platforms, YouTube(r) and WebSurg(r), in Sleeve Gastrectomy. Obes Surg.

[5] Lima DL, Cordeiro RN, Carvalho GL, Malcher F (2019). The influence of social media in minimally invasive surgery education How surgeons exchange experience and knowledge in these platforms. J Minim Access Surg.

[6] Myers CG, Kudsi OY, Ghaferi AA (2018). Social Media as a Platform for Surgical Learning Use and Engagement Patterns Among Robotic Surgeons. Ann Surg.

[7] Bernardi K, Milton AN, Hope W, Roth JS, Shah SK, Shah P (2020). Are online surgical discussion boards a safe and useful venue for surgeons to ask for advice A review of the International Hernia Collaboration Facebook Group. Surg Endosc.

[8] Jackson HT, Young MT, Rodriguez HA, Wright AS (2018). SAGES Foregut Surgery Masters Program a surgeon's social media resource for collaboration, education, and professional development. Surg Endosc.

[9] Rodriguez HA, Young MT, Jackson HT, Oelschlager BK, Wright AS (2018). Viewer discretion advised is YouTube a friend or foe in surgical education?. Surg Endosc.

[10] Toolabi K, Parsaei R, Elyasinia F, Zamanian A (2019). Reliability and Educational Value of Laparoscopic Sleeve Gastrectomy Surgery Videos on YouTube. Obes Surg.

[11] Kartal A, Kebudi A (2019). Evaluation of the Reliability, Utility, and Quality of Information Used in Total Extraperitoneal Procedure for Inguinal Hernia Repair Videos Shared on WebSurg. Cureus.

[12] Health Information Privacy (2015). HHS.gov.

[13] Bittner JG, Logghe HJ, Kane ED, Goldberg RF, Alseidi A, Aggarwal R (2019). A Society of Gastrointestinal and Endoscopic Surgeons (SAGES) statement on closed social media (Facebook(r)) groups for clinical education and consultation issues of informed consent, patient privacy, and surgeon protection. Surg Endosc.

[14] Wishner JD, Baker JW, Hoffman GC, Hubbard GW, Gould RJ, Wohlgemuth SD (1995). Laparoscopic-assisted colectomy The learning curve. Surg Endosc.

[15] Hung AJ, Chen J, Shah A, Gill IS (2018). Telementoring and Telesurgery for Minimally Invasive Procedures. J Urol.

[16] Vickers AJ, Bianco FJ, Gonen M, Cronin AM, Eastham JA, Schrag D (2008). Effects of pathologic stage on the learning curve for radical prostatectomy evidence that recurrence in organ-confined cancer is largely related to inadequate surgical technique. Eur Urol.

[17] Zorn KC, Gautam G, Shalhav AL, Clayman RV, Ahlering TE, Albala DM (2009). Training, credentialing, proctoring and medicolegal risks of robotic urological surgery recommendations of the society of urologic robotic surgeons. J Urol.

